# Burst activity and ultrafast activation kinetics of Ca_V_1.3 Ca^2+^ channels support presynaptic activity in adult gerbil hair cell ribbon synapses

**DOI:** 10.1113/jphysiol.2013.251272

**Published:** 2013-05-27

**Authors:** Valeria Zampini, Stuart L Johnson, Christoph Franz, Marlies Knipper, Matthew C Holley, Jacopo Magistretti, Sergio Masetto, Walter Marcotti

**Affiliations:** 1 Department of Biomedical Science, University of Sheffield Sheffield, S10 2TN, UK; 2 Department of Biology and Biotechnology, University of Pavia Pavia, 27100, Italy; 3 Department of Otolaryngology, THRC, Laboratory of Molecular Physiology of Hearing, University of Tübingen D-72076 Tübingen, Germany

## Abstract

Auditory information transfer to afferent neurons relies on precise triggering of neurotransmitter release at the inner hair cell (IHC) ribbon synapses by Ca^2+^ entry through Ca_V_1.3 Ca^2+^ channels. Despite the crucial role of Ca_V_1.3 Ca^2+^ channels in governing synaptic vesicle fusion, their elementary properties in adult mammals remain unknown. Using near-physiological recording conditions we investigated Ca^2+^ channel activity in adult gerbil IHCs. We found that Ca^2+^ channels are partially active at the IHC resting membrane potential (−60 mV). At −20 mV, the large majority (>70%) of Ca^2+^ channel first openings occurred with an estimated delay of about 50 μs in physiological conditions, with a mean open time of 0.5 ms. Similar to other ribbon synapses, Ca^2+^ channels in IHCs showed a low mean open probability (0.21 at −20 mV), but this increased significantly (up to 0.91) when Ca^2+^ channel activity switched to a bursting modality. We propose that IHC Ca^2+^ channels are sufficiently rapid to transmit fast signals of sound onset and support phase-locking. Short-latency Ca^2+^ channel opening coupled to multivesicular release would ensure precise and reliable signal transmission at the IHC ribbon synapse.

**Key points**

- The transfer of sound information to the brain relies on the precise release of neurotransmitter from sensory inner hair cell (IHC) ribbon synapses.
- Neurotransmitter release from IHCs is triggered by Ca^2+^ entry mainly through one type of Ca^2+^ channel (Ca_V_1.3).
- In this study we show that in near-physiological conditions Ca^2+^ channels open very rapidly following a stimulus with a delay of about 50 μs.
- Despite the open probability of the Ca^2+^ channels being very low, they can switch to a burst-like mode during a stimulus, maximizing Ca^2+^ influx into IHCs.
- These results help us to better understand how IHCs are able to accomplish high-fidelity signal transfer at individual auditory ribbon synapses.

## Introduction

The mammalian auditory system relies on temporally precise high-fidelity neurotransmitter release at inner hair cell (IHC) ribbon synapses (Fuchs, 2005). IHC neurotransmitter release is triggered by Ca^2+^ entry in response to cell depolarization during sound-induced hair bundle deflection. The IHC Ca^2+^ current is carried almost exclusively by Ca_V_1.3 Ca^2+^ channels (>90%: Platzer *et al.* 2000; Brandt *et al*. 2003), which are clustered at the presynaptic active zones and colocalized with readily releasable vesicles (Brandt *et al.* 2005; Graydon *et al.* 2011). However, the properties of Ca_V_1.3 Ca^2+^ channels in adult mammalian cells remain unknown and it is not clear whether their activation kinetics are sufficiently rapid to sustain phase locking to sound (Palmer & Russell, 1986).

In adult IHCs, a single ribbon synapse signals to an auditory afferent fibre, highlighting the importance of accurate neurotransmission at these synapses (Fuchs, 2005). In addition to ensuring sustained, high rates of vesicle release (Moser *et al.* 2006), hair cell ribbon synapses are able to synchronize the release of multiple vesicles to produce large AMPA-mediated excitatory postsynaptic currents (EPSCs; Glowatzki & Fuchs, 2002). The underlying mechanism for multivesicular release at ribbon synapses is unknown. Hair cell depolarization has been shown to increase the frequency and amplitude of EPSCs in lower vertebrates (Li *et al.* 2009). In mammals, only the frequency seems to be affected by IHC depolarization (Glowatzki & Fuchs, 2002; Grant *et al.* 2010). While there is no current explanation for the absence of EPSC amplitude increase with IHC depolarization, which is normally seen in other synapses (e.g. Christie & Jahr, 2006), their increase in frequency is thought to depend upon the incremental recruitment of Ca^2+^ channels per synapse with depolarization, with each new channel opening producing an additional vesicle fusion event (Brandt *et al.* 2005). The problem with this interpretation is that, classically, membrane depolarization is expected to increase the open probability of each Ca^2+^ channel and not the number of available Ca^2+^ channels. Therefore, upon depolarization each channel will be open for a longer time, which increases the probability of having overlapping channel openings. Here we analysed the unitary Ca_V_1.3 currents in adult mammalian IHCs to determine how they are likely to influence vesicle fusion at the presynaptic release site.

## Methods

### Ethics statement

In the UK, all animal studies were licensed by the Home Office under the Animals (Scientific Procedures) Act 1986 and were approved by the University of Sheffield Ethical Review Committee. In Germany, care and use of the animals and the experimental protocol were reviewed and approved by the animal welfare commissioner and the regional board for scientific animal experiments in Tübingen.

### Single hair cell electrophysiology

Basal-coil IHCs (frequency ∼30 kHz) from the adult gerbil were studied in acutely dissected cochleae (Johnson *et al.* 2008) from postnatal day 20 (P20) to P37, where the day of birth is P0. Gerbils (Charles River, UK) were killed by cervical dislocation. The cochleae were dissected in extracellular solution (in mm): 135 NaCl, 5.8 KCl, 1.3 CaCl_2_, 0.9 MgCl_2_, 0.7 NaH_2_PO_4_, 5.6 d glucose, 10 Hepes-NaOH, 2 sodium pyruvate, amino acids and vitamins (pH 7.5).

For single Ca^2+^ channel recordings, quartz glass patch pipettes were coated with surf wax (Mr Zoggs SexWax, USA) to minimize the fast electrode capacitative transient and filled with the following solution (in mm): 5 CaCl_2_, 102 CsCl, 10 Hepes-KOH, 15 4-aminopyridine (4 AP), 40 TEA (pH 7.5) containing linopirdine (100 μm; Tocris Bioscience, Bristol, UK), niflumic acid (50 μm; Sigma-Aldrich, Dorset, UK) and BayK 8644 (5 μm: Sigma). Stock solutions of niflumic acid and BayK 8644 were prepared in DMSO and stored at −20°C (final dilution 1:2000). Although the majority of recordings were performed using the above Na^+^-based extracellular solution, in a few experiments (Fig. 2*A* and *B*) the membrane potential of IHCs was zeroed by superfusing a high-K^+^ extracellular solution (Zampini *et al.* 2010) containing (in mm): 140 KCl, 0.2 CaCl_2_, 6.2 MgCl_2_, 0.7 NaH_2_PO_4_, 5.6 d-glucose, 15 Hepes-KOH (pH 7.5). Trypsin (0.025–0.05% v/v) was briefly and topically applied onto IHCs (40% of the recordings) prior to patching (Zampini *et al.* 2010). Data were filtered at 2 kHz or 5 kHz (4-pole Bessel) and sampled at 20 kHz or 50 kHz. Membrane potentials were corrected for the liquid junction potential (LJP) of +3 mV.

Whole-cell recordings were performed using soda glass capillaries (resistance 2–3 MΩ), coated with surf-wax and filled with (in mm): 106 caesium glutamate, 20 CsCl, 3 MgCl_2_, 1 EGTA-CsOH, 5 Na_2_ATP, 0.3 Na_2_GTP, 5 Hepes-CsOH, 10 sodium phosphocreatine (pH 7.3). The calcium current was isolated using extracellular TEA (30 mm), 4–AP (15 mm) and linopirdine (100 μm). Extracellular BayK 8644 (5 μm) and 5 mm Ca^2+^ were also used to mimic the single-channel recording conditions. Data were filtered at 5 kHz or 10 kHz (8-pole Bessel) and sampled at 50 kHz or 100 kHz. Membrane potentials were corrected for residual series resistance and LJP (−11 mV). All recordings were performed at body temperature (34–37°C).

### Immunocytochemistry

Gerbil cochleae (P20) were fixed (2% paraformaldehyde), decalcified and cryosectioned as previously described (Zampini *et al.* 2010). Primary antibodies to Ca_V_1.3 (rabbit, Alomone Laboratories, Jerusalem, Israel, diluted 1:50) and Ribeye/CtBP2 (mouse, BD Transduction Laboratories, Oxford, UK, diluted 1:50) were detected with Cy3-conjugated (Jackson ImmunoResearch Laboratories, West Grove, USA) or Alexa Fluor 488-conjugated (Life Technologies, Darmstadt, Germany) secondary antibodies. Images were acquired using a CCD camera and analysed with cellSense Dimension software (OSIS GmbH, Munster, Germany). The distribution of Ca^2+^ channels and ribbons were imaged over a distance of several micrometres with the coverage of the entire IHC synaptic region in an image-stack along the *z*-axis (*z*-stack) followed by three-dimensional deconvolution as previously described (Zampini *et al*. 2010). Immunolabelling was repeated at least three times in cells from different animals.

### Single Ca^2+^ channel data analysis

Single Ca^2+^ channel analysis was performed using Clampfit as previously described (Zampini *et al*. 2010). Briefly, leak and uncompensated capacitive currents were corrected by subtracting average episodes without channel activity (null sweeps) from the active sweeps. Event detection was performed with the 50% threshold detection method with each transition inspected before being accepted. Idealized traces were used to calculate channel amplitude distribution (event duration >0.34 ms), open probability (*P*_o_) and open and closed time histograms. Amplitude distributions were fitted with a Gaussian function. *P*_o_ was calculated as the fraction of time spent open *vs*. the total recording time. Sweeps containing two or more Ca^2+^ channels were excluded from the analysis. The total number of Ca^2+^ channels per IHC was estimated using:


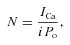
(1)

where *N* is the number of channels, *I*_Ca_ is the peak macroscopic Ca^2+^ current, *i* is the single-channel current size and *P*_o_ the channel open probability. To analyse the single channel open and closed times (Table 1), data from IHCs were pooled to obtain a distribution of dwell times on a log scale (12 bins decade^-1^) with normalization of the number of observations for bin amplitude. The plots obtained were interpolated, using the maximum likelihood method, with the sum of *n* (two or three) exponential functions (Zampini *et al*. 2010). The first latency distribution was investigated by measuring the time interval between the last point of the capacitative transient and the first opening. The distribution of the first latency was analysed as for the open and closed times. When fitting the dwell-time distributions, events of less than 0.34 ms in duration were ignored because they were under-represented due to low-pass filtering, which caused an underestimation of the fastest component of the first-latency distribution. Statistical comparisons of means were made using Student's two-tailed *t* test. Unless otherwise specified, mean values are quoted ±SEM, where *P* < 0.05 indicates statistical significance.

**Table 1:** Kinetic properties of single Ca^2+^ channels

A. First latency							
	τ_1_ (ms)	*W*_1_	τ_2_ (ms)	*W*_2_	τ_3_ (ms)	*W*_3_	No. of events
*V*_m_+ 50 mV (∼−20 mV)	0.18	73	6.3	18	201	9	39
B. Open time distribution							
	τ_o1_ (ms)	*W*_o1_	τ_o2_ (ms)	*W*_o2_	τ_o3_ (ms)	*W*_o3_	No. of events
*V*_m_+ 20 mV (∼−50 mV)	0.50	74	1.2	26			2199
*V*_m_+ 50 mV (∼−20 mV)	0.34	51	1.8	36	5.9	14	4652
C. Closed time distribution							
	τ_c1_ (ms)	*W*_c1_	τ_c2_ (ms)	*W*_c2_	τ_c3_ (ms)	*W*_c3_	No. of events
*V*_m_+ 20 mV (∼−50 mV)	0.40	14	7.0	33	113	53	2492
*V*_m_+ 50 mV (∼−20 mV)	0.71	89	7.6	9	95	2	4565

Time constants (τ) and the relative contributions (*W*, %) were obtained from the exponential fits of the latency of the first opening (A), open (B) and closed (C) time distributions at one or two different membrane voltages. Open (B) and closed (C) time constants obtained from fitting the dwell time distributions were grouped as follows: τ_1_ below 1 ms, τ_2_ between 1 ms and 10 ms, τ_3_ greater than 10 ms.

## Results

### Ca^2+^ channel distribution in IHCs along the adult gerbil cochlea

**Figure 1:**
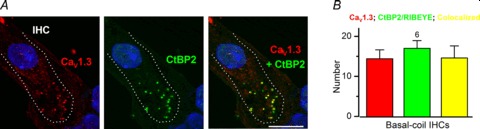
Distribution of Ca_V_1.3 and CtBP2/RIBEYE in adult gerbil IHCs *A*, basal-coil IHC from an adult (P20) gerbil cochlea immunostained for the Ca_V_1.3 Ca^2+^ channel (red) and ribbon marker CtBP2/RIBEYE (green). Colocalization is shown in the merge image in the right column. White dotted lines delineate IHCs. Images represent the maximum intensity projection over all layers of the *z*-stack. Nuclei were stained with DAPI (blue). Scale bar, 10 μm. *B*, total number of immunopositive spots for Ca_V_1.3 (red bar), total number of CtBP2/RIBEYE (green bar) and colocalized (yellow bar). Number of IHCs analysed for cochlear region is indicated above the bars.

In adult gerbil IHCs, Ca^2+^ channel clusters were only detected at the presynaptic region (Fig. 1*A*), which agrees with previous findings in apical-coil mouse IHCs (Brandt *et al.* 2005; Zampini *et al.* 2010). The average number of immuno-positive Ca_V_1.3 spots in basal IHCs was 14 ± 2 (*n*= 6), which were all colocalized with synaptic ribbons (CtBP2) (Fig. 1*B*). Despite performing cell-attached recordings from the bottom-half of IHCs, which contains the 14 presynaptic regions, the number of successful patches with stable Ca^2+^ channel activity was extremely low (∼4%).

### Unitary current of Ca_V_1.3 Ca^2+^ channels in adult IHCs

**Figure 2:**
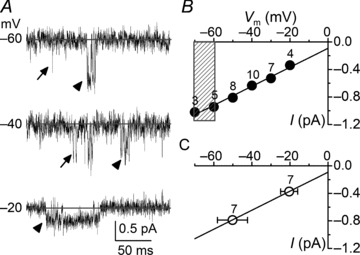
Ca^2+^ channel current in adult gerbil IHCs *A*, unitary Ca^2+^ currents recorded from adult gerbil IHCs using a high-K^+^ extracellular solution, and 5 mm Ca^2+^ and 5 μm BayK 8644 in the patch pipette. Transmembrane patch potentials are shown next to the traces. Grey horizontal lines indicate the channel closed state. Arrows and arrowheads show single brief and long-lasting clusters of openings, respectively. *B*, average current–voltage relation for single Ca^2+^ channel currents recorded in high-K^+^ extracellular solution (2 ≤*n*≤ 10 patches; 10 IHCs). Mean channel conductance: 14.7 ± 0.2 pS. Shaded area provides an indication for the resting membrane potential of adult IHCs. *C*, average single Ca^2+^ channel current amplitudes in Na^+^-based extracellular solution plotted on the fit from panel *B* in order to extrapolate the membrane potential (mean ± SD). Number of IHCs tested is shown. Unless otherwise stated, all recordings in this and the following figures were performed at 37°C.

Voltage-dependent L-type Ca^2+^ channels are encoded by four different pore-forming α1 subunit genes (α1C or Ca_V_1.2, α1D or Ca_V_1.3, α1S or Ca_V_1.1, α1F or Ca_V_1.4) and are sensitive to 1,4-dihydropyridines, such as the antagonist nifedipine and the agonist BayK 8644. In the mammalian cochlea, IHCs almost exclusively express the Ca_V_1.3 isoform (Platzer *et al*. 2000), and are therefore ideally suited to investigate the properties of this Ca^2+^ channel in isolation. Single Ca_V_1.3 Ca^2+^ channel recordings were performed from IHCs in acutely isolated cochleae maintained at 34–37°C, using 5 mm extracellular Ca^2+^ and 5 μm BayK 8644. The use of BayK 8644 was essential when working at 34–37°C since in its absence the majority of single-channel openings were not resolved and the apparent sub-conductive open states became very frequent. Although BayK 8644 is known to produce longer openings of L-type Ca^2+^ channel (Hess *et al*. 1984; Nowycky *et al*. 1985; Markwardt & Nilius 1988; Ceña *et al*. 1989), it does not affect the first latency (Hess *et al.* 1984), or its elementary Ca^2+^ conductance and voltage sensitivity (Zampini *et al*. 2010). As a result, at macroscopic level the impact of BayK 8644 is to increase the peak Ca^2+^ current about 3-fold with no change in activation kinetics (Zampini *et al.* 2010). Initially, experiments were performed in a high-K^+^ extracellular solution, which, by bringing the IHCs’ resting membrane potential near to 0 mV, allowed for control over transmembrane potential in the recorded patches (Zampini *et al.* 2010). Under these conditions, unitary Ca^2+^ channel openings became more frequent and longer lasting with membrane depolarization (Fig. 2*A*). Moreover, irrespective of the membrane potential (*V*_m_), Ca^2+^ channels exhibited two distinct opening modes: one characterized by brief and rather infrequent openings (arrows: Fig. 2*A*) and the other by long-lasting clusters or bursts of long and brief openings separated by brief closures (arrowheads: Fig. 2*A*). These two modes are reminiscent of gating ‘mode 1’ (brief) and ‘mode 2’ (long), previously reported for L-type Ca^2+^ channels (Hess *et al.* 1984; Nowycky *et al*. 1985). Although mode 2 gating is favoured by BayK 8644 (Hess *et al.* 1984; Nowycky *et al*. 1985), it is a characteristic behaviour of L-type Ca^2+^ channels. This is also indicated by the observation that clusters of brief and long openings (bursts) can be seen in the absence of BayK 8644, which increase the duration of all openings, in immature mouse IHCs (Zampini *et al.* 2010). Often, a specific gating mode was largely predominant in one or a group of successive sweeps, indicating that the two gating modes were not randomly distributed among sweeps, which is consistent with the idea that they are controlled by intracellular modulators (Nowycky *et al*. 1985; Kamp & Hell, 2000; Carabelli *et al.* 2001). The single-channel current–voltage (*I*–*V*) relation was linear in the voltage range investigated with an average slope conductance of 15 pS (Fig. 2*B* and *C*). This is similar to that measured in immature mouse IHCs (Zampini *et al.* 2010) and in a cell culture system (Bock *et al.* 2011), but larger than that proposed for frog hair cells (3.5 pS: room temperature, 2 mm Ca^2+^, Graydon *et al.* 2011). We found that single Ca^2+^ channel activity was present at the resting *V*_m_ for adult IHCs (−60 mV: Johnson *et al*. 2011), and fell within the activation range of the macroscopic *I*_Ca_ (Johnson *et al*. 2008).

### Ca_V_1.3 Ca^2+^ channel open probability in Na^+^-based extracellular solution

**Figure 3:**
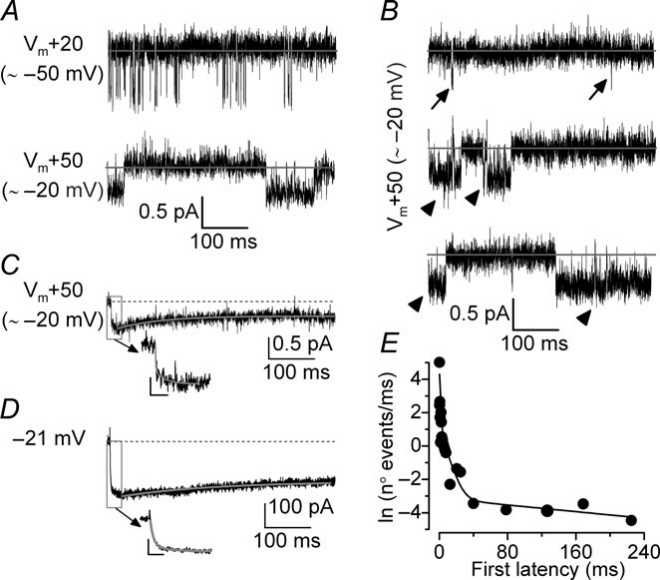
Ca^2+^ channel currents recorded in Na^+^-based solution *A*, representative unitary currents recorded from basal IHCs in a Na^+^-based extracellular solution with 5 mm Ca^2+^ and 5 μm BayK 8644. *B*, examples of channel openings near −20 mV with mode 1 (brief: arrow) and mode 2 (bursts: arrowhead). Grey horizontal lines in *A* and *B* indicate the channel closed state. *C*, ensemble-averaged Ca^2+^ current near −20 mV. The value of the scale bars in the expanded time course of activation are: 0.3 pA and 10 ms. In *A*–*C* the patch transmembrane voltage is indicated as the unknown IHC *V*_m_ plus the voltage step delivered to the patch pipette (e.g. *V*_m_+ 20 mV: 20 mV depolarization from *V*_m_). The actual estimated patch transmembrane voltage is shown in parentheses (see Results). *D*, macroscopic *I*_Ca_ at −21 mV recorded from a basal IHC using the same Na^+^-based extracellular solution to that used for single-channel recordings. Scale bars near the expanded time course of activation of the single-channel (*C*) and whole-cell (*D*) currents are: 100 pA and 5 ms. Superimposed lines in *C* and *D* are single exponential fits (activation is also shown on an expanded timescale). *E*, first latency distribution was obtained by plotting the natural logarithm of the number of observations ms^-1^ (Zampini *et al.* 2010) as a function of latency.

We investigated single Ca^2+^ channel properties while maintaining IHCs at their physiological *V*_m_ using a Na^+^-based extracellular solution. The Na^+^-based solution prevented us from directly determining the IHC resting *V*_m_ in cell-attached recordings. Therefore, the patch transmembrane voltage is indicated as the unknown IHC *V*_m_ plus the voltage step delivered to the patch pipette (e.g. *V*_m_+ 20 mV: 20 mV depolarization from *V*_m_). The actual patch transmembrane voltage was estimated using the amplitude of the elementary Ca^2+^ current and extrapolating it from the *I*–*V* curves obtained in high-K^+^ solution (Fig. 2*B* and *C*), assuming identical single-channel conductance between the two recording conditions (Zampini *et al.* 2010). Calcium channel recordings obtained by applying 500 ms step depolarizations to *V*_m_+ 20 mV and *V*_m_+ 50 mV in a Na^+^-based solution are shown in Fig. 3*A*. The estimated transmembrane voltage applied to IHCs was about −50 mV for *V*_m_+ 20 mV and −20 mV for *V*_m_+ 50 mV (Fig. 2*C*). Calcium channel gating ‘mode 1’ and ‘mode 2’ (Fig. 2*A*) were also seen in the Na^+^-based solution (Fig. 3*B*; see also Supplemental Fig. 1, available online only).

The percentage of null-sweeps in adult IHCs was 46% near −20 mV. When only sweeps containing channel openings (500 ms duration) were considered, the Ca^2+^ channel mean open probability (*P*_o_) increased with depolarization from 0.01 at about −50 mV to 0.21 at about −20 mV. We found that the maximal *P*_o_ varied significantly among sweeps, from the lowest value of 0.014 when the channel opened rarely and briefly (gating mode 1) to 0.91 in the presence of prolonged periods of opening (mode 2). The total number of Ca^2+^ channels present in adult IHCs (see eqn (1): *I*_Ca_=−197 pA; *i*=−0.34 pA; *P*_o_= 0.21) is likely to be in the order of 2800. The macroscopic *I*_Ca_ (−197 pA) was measured in adult gerbils using experimental conditions similar to those used for single-channel recordings (see Methods). A higher *P*_o_ of ∼0.8 and smaller elementary conductance has previously been estimated using fluctuation analysis by calculating the variance and mean of whole-cell tail Ca^2+^ currents measured at −62 mV from pre-step depolarization to +58 mV (Brandt *et al.* 2005). However, strong membrane depolarization (+58 mV), as opposed to voltage levels within a physiological range (−20 mV), has been shown to produce an increased frequency of long-duration (mode 2) Ca^2+^ channel openings (Josephson *et al.* 2002), which will result in *P*_o_ overestimation. Despite the presence of BayK 8644 in our recording conditions, a similar or slightly higher Ca^2+^ channel *P*_o_ was also found in bullfrog hair cells (Graydon *et al.* 2011) and in the retina (Doering *et al.* 2005), indicating that the low *P*_o_ is likely to be a characteristic of Ca^2+^ channels at ribbon synapses. Ca_V_1.3 Ca^2+^ channel splice variants with very low *P*_o_ have also been described in cell culture systems (Bock *et al.* 2011).

### Kinetic properties of the Ca^2+^ current

The activation and inactivation time constants of the ensemble-average current (Fig. 3*C*: τ_activation1_= 0.33 ms, τ_activation2_= 6.08 ms, τ_inactivation_= 92 ms; 300 sweeps from 8 IHCs) from single-channel recordings (Supplemental Fig. 1) were in the range of those obtained with whole-cell recordings (Fig. 3*D*: τ_activation1_= 0.50 ± 0.03 ms, τ_activation2_= 3.33 ± 0.56 ms, τ_inactivation_= 151 ± 15 ms, *n*= 5) using similar experimental conditions (5 mm Ca^2+^, BayK 8644). The similarity between the kinetics of the ensemble-average current and that recorded in whole-cell suggests that the cell-attached configuration, and the mechanical perturbation it could induce upon the patched membrane, is unlikely to significantly alter the kinetic behaviour of the Ca^2+^ channels. Using whole-cell recordings, we also found that the activation kinetics of *I*_Ca_ in 5 mm Ca^2+^ (0.74 ± 0.22 ms near −10 mV, *n*= 5) were about 4 times slower compared to those in 1.3 mm Ca^2+^ (0.16 ± 0.02 ms, *n*= 7), which is most likely caused by surface screening effects (Byerly *et al.* 1985; Smith *et al.* 1993).

The distribution of first latencies, the delay between the stimulus onset and the first observed Ca^2+^ channel opening, was well defined by the sum of three exponentials (Fig. 3*E*). The fastest component showed a sub-millisecond time constant near the peak of the macroscopic *I*_Ca_ (−20 mV), the weight of which was much larger than the other two components (Table 1). The fast component's relative weight is likely to be greater than our estimate, since in several sweeps (28%) containing early-onset channel openings the first latencies could not be measured, due to the residual capacitive transient (see Methods). The similarity between the first two time constants of the first latency distribution (τ_1_= 0.18 ms; τ_2_= 6.3 ms in 5 mm Ca^2+^; Table 1) and those of the macroscopic *I*_Ca_ activation, indicates that they are the main determinant of current activation in response to membrane depolarization. Since in the presence of 1.3 mm extracellular Ca^2+^ the activation kinetics of macroscopic *I*_Ca_ became about 4 times faster (see above), the first latency time constants can also be expected to become faster. We estimate that τ_1_, which represents 73% of the total first latency distribution (Table 1), would decrease from 0.18 ms to about 50 μs.

**Figure 4:**
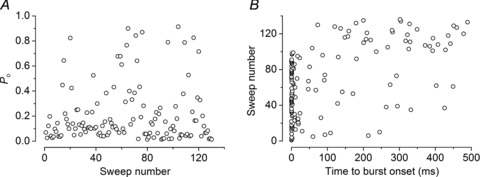
Single Ca^2+^ channel bursting activity in adult IHCs decreases during a sweep *A*, calcium channel *P*_o_ at around −20 mV as a function of successive sweeps from basal IHCs. *B*, time of burst onset obtained at around −20 mV. Note that bursts appear more frequently at the very beginning of the sweep.

Fitting the dwell time distributions (data not shown) revealed two or three open (τ_o1_, τ_o2,_τ_o3_) and three closed (τ_c1_, τ_c2_, τ_c3_) time constants (Table 1). We found that depolarization induced an overall increase in the relative weight of τ_o2_, the appearance of a longer time constant (τ_o3_) and an increase in the weight of the shortest close time constant (τ_c1_). A very slow exponential component, with a time constant (τ_c3_) of about 95 ms, was also present in closed-time distributions. Although τ_c3_ was probably underestimated, due to the high probability of long closure events being interrupted at the end of the 500 ms depolarization, it was 12 times greater than the ‘intermediate’ time constant τ_c2_. Moreover, the relative weight of the slowest component was only 2%. Therefore, the average number of ‘short’ closures per sweep exceeded that of ‘long’ closures, indicating that single Ca^2+^ channel openings had a relatively high probability of being separated by short closings. This implies that Ca^2+^ channel activity was largely organized in bursts, consisting of sequences of openings separated by short closings, and interrupted by prolonged closures (Fig. 3*A* and *B*). The mean burst duration, defined as any cluster of openings occurring without superimpositions and separated from the previous and/or following openings by an interval of at least 15 ms (i.e. twice the value of τ_c2_), was 81 ± 72 ms (136 bursts from 101 sweeps: 8 IHCs). Bursting activity greatly increased *P*_o_ in a sweep. As seen with the high-K^+^ extracellular solution, bursts of channel openings often appeared in successive sweeps (Fig. 4*A*), indicating a shift of the Ca^2+^ channel gating mode towards bursts with depolarization. Moreover, burst onsets were concentrated at the very beginning of the sweep (Fig. 4*B*), consistent with the short Ca^2+^ channel first latency.

## Discussion

In this study, using near-physiological experimental conditions, we determined that in adult IHCs the first Ca^2+^ channel opening latency following membrane depolarization is likely to be about 50 μs. We also found that most Ca^2+^ channel openings are rare and very brief (∼0.5 ms). Despite the low mean *P*_o_, Ca^2+^ influx into IHCs through a Ca^2+^ channel can be maximized by burst activity. We propose that brief single Ca^2+^ channel openings are sufficient to trigger vesicle release, and the short-latency would ensure reliable and precise signal transmission at the IHC ribbon synapse during high-frequency activity.

### Ca^2+^ channel short latency allows high-frequency tuning

The coding of auditory stimuli in mammals requires temporally precise transfer at IHC ribbon synapses (Fuchs, 2005). However, the activation kinetics of IHC Ca^2+^ channels were deemed to be too slow (several milliseconds in adult hair cells: reviewed by Moser *et al.* 2006) to follow the exact timing of sound stimuli. We found that the fastest time constant of Ca^2+^ channel first latency (τ_1_) was ∼0.18 ms in adult gerbil IHCs (at −20 mV; 5 mm Ca^2+^; 34–37°C). This value is much faster than that recorded in immature mouse IHCs (τ_1_, 0.70 ms: Zampini *et al.* 2010), indicating that the kinetic properties of Ca^2+^ channel change with development similar to the macroscopic *I*_Ca_ (Johnson & Marcotti, 2008). Moreover, the fast component of the total first latency distribution was predominant, contributing about 73% of it (Table 1). Finally, the comparison with whole-cell recordings indicated that in the presence of 1.3 mm Ca^2+^, real τ_1_ values can be assumed to be even smaller at any given potential, with an expected value of 50 μs at −20 mV (see Results). These findings show that Ca^2+^ channel activation rates are sufficiently rapid to support phase-locking to sound (Palmer & Russell, 1986).

### Single Ca^2+^ channel openings sustain release at rest

IHCs release glutamate tonically, modulating the rate of release as stimulus intensity changes. The resting *V*_m_ of adult IHCs has been estimated *in vitro* to be around −60 mV using physiological conditions (Johnson *et al.* 2011). Since we observed Ca^2+^ channel activity at membrane potentials as negative as −70 mV (Fig. 1), a fraction of these channels is likely to be active at rest. The resting Ca^2+^ channel activity would elicit ‘spontaneous’ neurotransmitter release at IHC ribbon synapses and drive the background firing activity observed in auditory afferent fibres (Robertson & Paki, 2002). We calculated that there are about 2800 Ca^2+^ channels present in adult IHCs, which is similar to that found in immature mouse IHCs (Zampini *et al.* 2010) and adult bullfrog hair cells (Rodriguez-Contreras & Yamoah, 2001; Graydon *et al.* 2011). The similar elementary conductance of Ca^2+^ channels recorded from different patches in gerbil IHCs is consistent with previous findings showing that in these cells the Ca^2+^ current is almost exclusively carried by Ca_V_1.3 channels (Platzer *et al.* 2000; Brandt *et al.* 2003). Recent fast confocal Ca^2+^ imaging studies have hypothesized that IHCs could adjust the number and the gating of Ca_V_1.3 channels at their active zones to diversify their transmitter release rates (Frank *et al.* 2009). This is consistent with our observation that the gating (mode) of Ca_V_1.3 channels varies significantly in the same patch, presumably as a consequence of intracellular modulation. The presence of different Ca_V_1.3 splice variants and/or intracellular modulators among different synapses could allow IHCs to regulate neurotransmitter release at distinct active zones.

Considering that high-frequency adult gerbil IHCs contain ∼14 active zones, and assuming that in these cells ∼2800 Ca^2+^ channels are expressed, ∼10% of which seem to be extra-synaptic (Meyer *et al.* 2009), then there would be ∼180 Ca^2+^ channels in each presynaptic active zone (∼0.25 μm^2^: Lenzi & von Gersdorff, 2001; Meyer *et al.* 2009). This high channel density agrees with that reported for Ca_V_2.1 channels in hippocampal glutamatergic terminals (Holderith *et al*. 2012) and for Na^+^ channels in rapidly conducting systems such as the rat node of Ranvier (Neumcke & Stämpfli, 1982). Given the mean single-channel *P*_o_ of 0.01 at −50 mV, on average two Ca^2+^ channels per active zone would be simultaneously open near the IHC resting *V*_m_. However, considering that Ca^2+^ channel bursting (mode 2) is the main contributor to the *P*_o_, and that in the absence of BayK 8644 the channel *P*_o_ is expected to be even lower, it is likely that a single-channel opening will provide enough Ca^2+^ to trigger a vesicle fusion event, which is in partial agreement with previous indirect observations (Brandt *et al.* 2005; Li *et al.* 2009).

An interesting feature of hair cell ribbon synapses is that vesicle release evokes short-lived EPSCs of variable amplitude (Glowatzki & Fuchs 2002; Li *et al.* 2009; Grant *et al.* 2010), the majority (>70%) of which show a rapid monophasic rise time (<1 ms: Li *et al.* 2009; Grant *et al.* 2010). Large monophasic EPSCs are thought to originate from a highly synchronized and very rapid presynaptic fusion of multiple vesicles (up to 20). Currently, the mechanism underlying multivesicular release in IHCs is unknown. Given the very low Ca^2+^ channel resting *P*_o_ in IHCs and other ribbon synapses (Graydon *et al.* 2011; Doering *et al.* 2005), it is extremely unlikely that large monophasic EPSCs originate from simultaneous opening of distinct Ca^2+^ channels (estimated probability for two simultaneous openings is ∼0.3% or even lower without BayK 8644). Thus, our gerbil IHC data indicate that a single Ca^2+^ channel opening is likely to be able to trigger the simultaneous fusion of multiple vesicles, which supports a similar proposal for bullfrog hair cells (Graydon *et al.* 2011). At retinal bipolar cell ribbon synapses, it has been proposed that multivesicular release could originate from the compound fusion of multiple vesicles (Sterling & Matthews, 2008). In hair cells, evidence for such a mechanism remains elusive. Recent findings showed that the amplitude of EPSCs is independent of presynaptic Ca^2+^ influx (Glowatzki & Fuchs, 2002; Grant *et al.* 2010), indicating that postsynaptic mechanisms could also contribute to the highly variable EPSC amplitude, as proposed for central glutamate synapses (Franks *et al.* 2003). Indeed, the density of postsynaptic AMPA receptors has been shown to vary at a single IHC ribbon synapse (Ottersen *et al.* 1998; Meyer *et al.* 2009) as well as among different afferent terminals (Liberman *et al.* 2011).

### Single Ca^2+^ channel openings during membrane depolarization

During sound-induced stimulation, the IHC receptor potential is driven by the mechanotransducer current. We found that membrane depolarization greatly increased the open probability of Ca^2+^ channels: *P*_o_ changed from 0.01 at rest to 0.21 at −20 mV. This agrees with the observation that the frequency of EPSCs increases with IHC depolarization (Glowatzki & Fuchs, 2002). However, there are two unusual features of EPSC recordings that are difficult to reconcile with the single Ca^2+^ channel properties: (1) EPSC amplitude does not increase with IHC depolarization, which is normally seen in other synapses (e.g. Christie & Jahr, 2006), indicating that their size is independent of the amount of Ca^2+^ influx into the cell (Glowatzki & Fuchs, 2002; Goutman & Glowatzki, 2007); (2) monophasic EPSCs remain more frequent than multiphasic EPSCs with IHC depolarization (Li *et al.* 2009; Grant *et al.* 2010), which contradicts what we would anticipate considering that the increased Ca^2+^ channel *P*_o_ with depolarization is expected to produce mostly random, non-synchronized, overlapping channel openings.

Pharmacological manipulation of the macroscopic Ca^2+^ influx into IHCs led to the hypothesis that large EPSCs could originate from the incremental recruitment of single Ca^2+^ channels with depolarization (Brandt *et al.* 2005). However, adult IHCs express a homogeneous population of Ca_V_1.3 Ca^2+^ channels, with analogous voltage dependency, and depolarization only increased the *P*_o_ of each Ca^2+^ channel, and not the number of available Ca^2+^ channels: in a single patch, Ca^2+^ channel *P*_o_ varied from <0.01 at −50 mV to >0.9 at −20 mV with no sign of overlapping Ca^2+^ channels. This means that all Ca^2+^ channels controlling vesicle fusion, which are presumably equally sensitive to voltage change, will, on average, be open for longer with membrane depolarization. Since Ca^2+^ channel *P*_o_ is largely determined by gating mode 2 (bursting), we propose instead that depolarization increases the chance that Ca^2+^ channels opening in mode 1 (largely silent) switch to mode 2 (bursting), thus increasing the probability of vesicle-fusion events. This is likely to be true even in the absence of BayK 8644 since Ca_V_1.3 Ca^2+^ channels show bursting behaviour even without the agonist (Zampini *et al*. 2010). Macroscopically, this would appear as an apparent increase in the number of active Ca^2+^ channels (Brandt *et al.* 2005). This model is consistent with the observation that the frequency of EPSCs of varying amplitude increases with IHC depolarization (Glowatzki & Fuchs, 2002). On the other hand, the presence of the low incidence of multiphasic EPSCs (about 30%: Grant *et al.* 2010) could result from Ca^2+^-induced Ca^2+^ release from intracellular stores or perhaps be due to Ca^2+^ occasionally escaping from the nanodomain and diffusing to additional release sites. This ‘spillover’ of Ca^2+^ ions could be a consequence of prolonged Ca^2+^ channel openings during bursts of activity and the saturation of the Ca^2+^ sensor(s) at the presynaptic site, or saturation of intracellular Ca^2+^ buffers, since its volume seems to be restricted by the presence of the ribbon (Graydon *et al.* 2011).

## Additional information

### Author's present address

V. Zampini: Département de Biologie, École normale supérieure, Paris, France.
