# Supplementary material for: Burst activity and ultrafast activation kinetics of CaV1.3 Ca2+ channels support presynaptic activity in adult gerbil hair cell ribbon synapses
Source: J Physiol. 2013 May 27;591(Pt 16):3811–20. doi: 10.1113/jphysiol.2013.251272 (PMC3764630; doi:10.1113/jphysiol.2013.251272)
Supplement: Supplementary file 1 [file tjp0591-3811-SD1.pdf]

Burst activity and ultrafast activation kinetics of Ca_V1.3 Ca^{2+} channels support presynaptic activity in adult gerbil hair cell ribbon synapses

Valeria Zampini, Stuart L. Johnson, Christoph Franz, Marlies Knipper, Matthew C. Holley, Jacopo Magistretti, Sergio Masetto and Walter Marcotti

This document includes Supplementary Figure 1.

Supplementary Figure 1

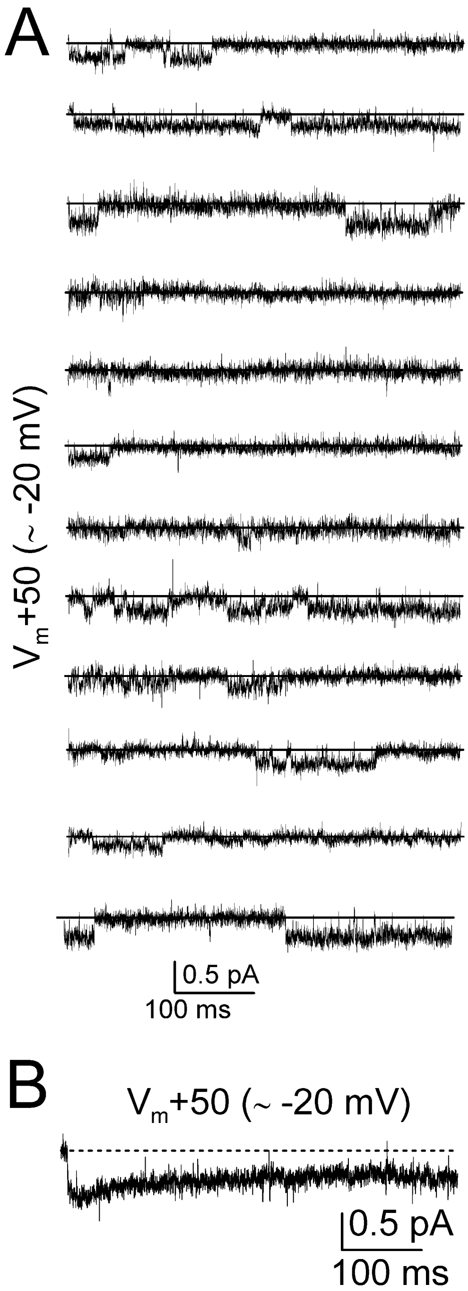

Supplementary Figure 1. Single Ca^{2+} channel currents.

A, Representative unitary Ca^{2+} currents near –20 mV with mode 1 and mode 2, including those showed in Figure 3*B*. Recordings are from basal IHCs in a Na^{+}-based extracellular solution with 5 mM Ca^{2+} and 5 μM BayK 8644. Grey horizontal lines indicate the channel closed state. ***B***, Ensemble-averaged as shown in Figure 3C.
